# Histopathological assessments reveal retinal vascular changes, inflammation, and gliosis in patients with lethal COVID-19

**DOI:** 10.1007/s00417-021-05460-1

**Published:** 2021-10-29

**Authors:** Vijay K. Jidigam, Rupesh Singh, Julia C. Batoki, Caroline Milliner, Onkar B. Sawant, Vera L. Bonilha, Sujata Rao

**Affiliations:** 1 Department of Ophthalmic Research, Cole Eye Institute, Cleveland Clinic, 9500 Euclid Avenue, Cleveland, OH 44195 USA; 2 Center for Vision and Eye Banking Research, Eversight, 6700 Euclid Ave, Suite 101, Cleveland, OH 44103 USA; 3 Department of Ophthalmology, Cleveland Clinic Lerner College of Medicine of Case Western Reserve University, Cleveland, OH 44195 USA

**Keywords:** COVID-19, Retina, Histopathology, Vasculature, Inflammation

## Abstract

**Purpose:**

The purpose of this study is to assess for histopathological changes within the retina and the choroid and determine the long-term sequelae of the SARS-CoV-2 infection.

**Methods:**

Eyes from seven COVID-19-positive and six similar age-matched control donors with a negative test for SARS-CoV-2 were assessed. Globes were evaluated ex vivo with macroscopic, SLO and OCT imaging. Macula and peripheral regions were processed for Epon embedding and immunocytochemistry.

**Results:**

Fundus analysis shows hemorrhagic spots and increased vitreous debris in several of the COVID-19 eyes compared to the controls. OCT-based measurements indicated an increased trend in retinal thickness in the COVID-19 eyes; however, the difference was not statistically significant. Histology of the retina showed presence of hemorrhages and central cystoid degeneration in several of the donors. Whole mount analysis of the retina labeled with markers showed changes in retinal microvasculature, increased inflammation, and gliosis in the COVID-19 eyes compared to the controls. The choroidal vasculature displayed localized changes in density and signs of increased inflammation in the COVID-19 samples.

**Conclusions:**

In situ analysis of the retinal tissue suggests that there are severe subclinical abnormalities that could be detected in the COVID-19 eyes. This study provides a rationale for evaluating the ocular physiology of patients that have recovered from COVID-19 infections to further understand the long-term effects caused by this virus.
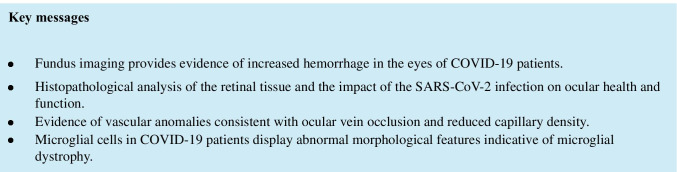

**Supplementary Information:**

The online version contains supplementary material available at 10.1007/s00417-021-05460-1.

## Introduction

We are amid the human coronavirus disease 2019 (COVID-19) pandemic, caused by severe acute respiratory syndrome coronavirus (SARS-CoV-2), which is of historic proportions, the likes of which we have not seen in 102 years. With >25 million cases confirmed, > 490 K deaths in the USA (WHO COVID-19 Dashboard), it is one of the deadliest events in US history, and rates continuing to rise, the end is not in near sight. Despite being primarily a respiratory virus, COVID-19 can also present with non-respiratory signs, including ocular symptoms as conjunctival hyperemia, chemosis, epiphora, increased secretions, ocular pain, photophobia, and dry eye [1–6]. SARS-CoV-2 requires host cellular receptors (such as ACE2) for successful replication during infections. Immuno-histochemical studies and single-cell RNA-sequencing datasets have revealed both extra- and intra-ocular localization of SARS-CoV-2 receptors, ACE2 receptor, and TMPRSS2 protease in human eyes [1, 7–9]. The virus has also been detected within the anterior chamber and in the ocular fluids suggesting that ocular tissue may be directly affected due to Sars-CoV-2 infection [1, 10, 11]. Evidence of posterior eye involvement in SARS-COV-2 infection is still scarce, though some recent optical coherence tomography angiography (OCTA)-based findings show that retinal microvasculature is affected in patients that recovered from COVID-19 infection [12, 13]. However, a detailed histopathological analysis of the retinal tissue and the impact of the SARS-CoV-2 infection on ocular health and function have not been examined. Here we report a comprehensive analysis of eyes from post-mortem patients infected with the SARS-CoV-2 virus. Fundus imaging provides evidence of increased hemorrhage in the eyes of COVID-19 patients. In some SARS-CoV-2-positive patients, there was evidence of vascular anomalies consistent with ocular vein occlusion and reduced capillary density. Additionally, there is an overall increase in the number of microglial cells within the retina of COVID-19 patients. The microglial cells display abnormal morphological features indicative of microglial dystrophy. There is also increased gliosis in the COVID-19 eyes compared to the eyes from the age-matched control donors. To our knowledge, this is the first study that provides in vivo molecular information concerning the changes occurring within the retinal tissue of COVID-19 patients. We are still in the midst of the coronavirus outbreak, and there is constantly emerging information regarding its long-term sequelae on various systems in the body. The data reported here provide a rationale for longitudinal ocular assessments in recovered patients to truly gain insights into understanding the long-term effects caused by this virus.

## Materials and methods

### Tissue acquisition and fixation

**Fig. 1:**
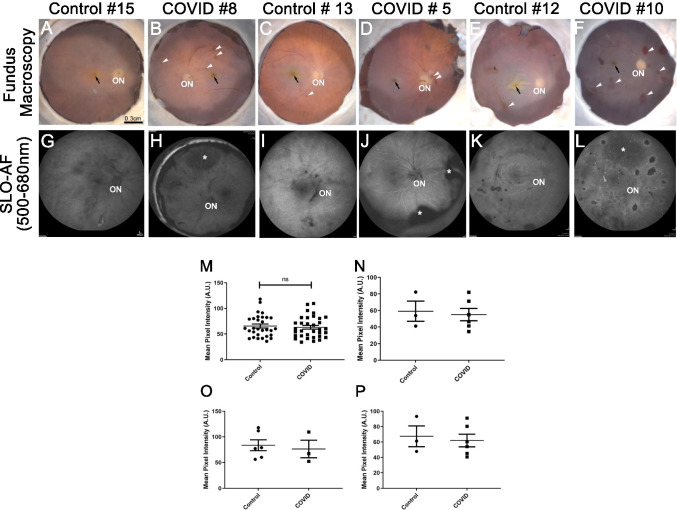
Ex vivo imaging of COVID-19 donor eyes. * Representative fundus (**A–F**) and SLO images (**G–L**) collected from COVID-19 and age-similar controls. Fovea (black arrow) and optic nerve head (ON) were visible in all eyes. Hemorrhage spots (white arrowheads) were visible in most of the COVID-19 eyes. BAF images of COVID-19 (**H, J, L**) and control (**G, I, K**) eyes revealed a pattern that matched the fundus images. Also, in the COVID-19 eyes, the detached retinas were apparent with SLO (**H, J, L,** *). Mean intensity calculation of the OD and OS eyes, using BAF SLO images from all age groups (**M**), age group 90 (**N**), age group 75 (**O**), and age group 46/35 (**P**). Scale bar **A**–**F** = 0.3 cm, **G–L** = 200 μm

Donor eyes were obtained through Eversight (Cleveland, OH). Eye bank records accompanying the donor eyes indicated whether the donor had COVID-19. Pathology analysis was performed with the approval of the Cleveland Clinic Institutional Review Board (IRB #20-755) and Institutional Biosafety Committee (IBC# 2018). The research adhered to the tenets of the Declaration of Helsinki. Tissue from seven COVID-19 and six control donors was analyzed. Three of the control donors (# 11, 15, 16) were kept on ventilators prior to their death. Control #14 displayed cardiomegaly with vascular congestion. Control donors #11, 12, 13, 14 had past history medical history of cerebrovascular accident/stroke/transient ischemic attack. Oxymetric readings for all controls and COVID-19 patients prior to their death are between 93 and 95%. Additional information about the donors is provided in Table 1 (Fig. 1). Cornea and anterior segment analysis for the COVID-19 donors were recently reported by Sawant et al. [11]. In this study, Case#1 and Case#9 were excluded from the analysis as the eyes were damaged during enucleation and their retinas could not be analyzed. For analysis, globes without the cornea were fixed and kept in 4% paraformaldehyde and 0.5% glutaraldehyde made in Dulbecco’s phosphate-buffered saline (D-PBS) buffer for at least a month.

**Table 1:** Human donor information

CASE no^a^	Age^b^	Gender^c^	Race^d^	PMI^e^	SARS-Cov-2 testing	Hospitalization days	Cause of death
**2**	89	F	C	12	Positive^f^	2	COVID-19, pneumonia, respiratory failure
**4**	35	M	SA	7	Positive^f^	72	COVID-19, respiratory failure, ICH
**5**	75	F	C	27	Positive^f^	26	COVID-19, respiratory failure
**6**	67	M	H	20	Positive^f^	11	COVID-19, respiratory failure
**7**	59	F	C	11	Positive^f^	15	Severe hypotension secondary to septic shock
**8**	90	F	H	16	Positive^f^	5	COVID-19, respiratory failure
**10**	46	M	H	13	Positive^f^	45	COVID-19, respiratory failure
**11**	78	M	C	5	Negative	5	Stroke
**12**	36	F	C	17	Negative	1	Chronic kidney disease
**13**	75	M	C	7	Negative	12	Myocardial infarction
**15**	90	M	C	24	Negative	6	Myocardial infarction
**16**	63	F	C	13	Negative	40	End stage liver disease
**17**	61	M	C	7	Negative	3	Stroke, bladder cancer

^a^Cornea and anterior segments analysis were reported in Sawant et al., *Ocul Surf.* 2020 Nov 8;S1542-0124(20)30168-3. 10.1016/j.jtos.2020.11.002

^b^Age: age at death (years)

^c^Gender: *M* = male, *F* = female

^d^Race: *AA* = African American, *C* = Caucasian, *H* = Hispanic, *SA* = South Asian

^e^Interval from death to preservation (hours)

^f^Results reported in Sawant et al., *Ocul Surf.* 2020 Nov 8;S1542-0124(20)30168-3. 10.1016/j.jtos.2020.11.002

### Ex vivo imaging of globes

Globes were cut through the ora serrata, and posterior poles were transferred to a chamber filled with D-PBS solution and imaged as previously described [14]. Briefly, fundus macrophotography [15] images were collected using a Zeiss AxioCam MRC5 camera equipped with a macro zoom lens and AxioVision AC Software (Zeiss). Fundus autofluorescence was obtained using blue autofluorescence (BAF) mode of Heidelberg Spectralis confocal scanning laser ophthalmoscopy (SLO) (Heidelberg Engineering, Inc.). Optical coherence tomography images (OCT) were collected using spectral domain OCT system (Envisu R2210 UHR Leica Microsystems Inc.). Both the OD and OS eyes were imaged (see Supplemental Fig. S1).

### Histopathology

Fragments of retina-RPE-choroid were cut from the periphery to the optic nerve head and placed in 2.5% glutaraldehyde in 0.1 M cacodylate buffer, sequentially dehydrated in ethanol and embedded in Epon as previously reported [16]. For light microscopy, semi-thin sections were cut with a diamond histotech knife, dried, and stained with toluidine blue. Slides were photographed with a Zeiss AxioImager.Z1 light microscope, and the images were digitized using a Zeiss AxioCam MRc5 camera. Only the OD eyes were used for the histopathology analysis.

### Retinal flatmount and immunohistochemistry

A piece of retina and choroid were cut from the posterior pole of each eyecup. The retina was dissected and washed overnight in TBS. The RPE/choroid was incubated in the disodium salt of ethylenediaminetetraacetic acid for 1.5 h, and RPE was removed using a pipette before washing with TBS overnight, and wholemount staining was performed as described with the exception that the tissues were incubated with the antibodies and the UEA lectin for 4 days [17]. Retinas were stained with chicken anti-glial fibrillary acidic protein (GFAP; 1:500; Millipore, Burlington, MA, USA), mouse anti-SARS-CoV S Protein (1:100; NR-614, BEI Resources, NIAID, Manassas, VA, USA), rabbit anti-Iba-1 (1:250; Wako Chemicals USA, Inc., Richmond, VA), and *Ulex europaeus* agglutinin-FITC (UEA lectin1:100; GeneTex, Irvine, CA, USA). Choroids were stained with UEA lectin and Iba-1 antibody. The submacular regions of the retina were imaged using a Leica confocal microscope. Retinal images were acquired from three different zones, one near the ONH and Vein, one near the middle, and one towards the periphery [18, 19]. All care was taken to ensure that similar regions were represented in the images. The choroids were imaged with Bruch’s membrane proximal to the objective.

### Retinal Western blots

Upon receiving posterior poles were cut through the ora serrata, vitreous was removed. Retinas were mechanically detached from the RPE, cut into smaller pieces, and fresh frozen for later analysis. Samples were lysed in lysis buffer composed of 20 mM Tris-HCl, pH 8, 150 mM NaCl, 2.5 mM EDTA, 10% glycerol, 0.5% Triton X-100, 0.01% Nonidet P-40 substitute, and protease inhibitor cocktail tablets (Roche Diagnostics, Indianapolis, IN, USA) and transferred to PVDF membranes (Immobilon- FL; Merck Millipore; Burlington). Membranes were probed with antibodies against ACE2 (1:1000, EPR4435(2), ab108252; Abcam, Cambridge, MA, USA) and TMPRSS2 (1:1000, EPR3861, ab92323, Abcam) followed by washing and incubation with anti-rabbit IRDye®680RD and anti-rabbit IRDye®800CW, (all from LI-COR Biosciences, Lincoln, NE). Immunoreactive signals were visualized using Odyssey CLx (LI-COR Biosciences).

### Vessel density and GFAP intensity quantification

Lectin-positive field images (0.33 mm^2^) from each region (from ONH/V, Mid/V, and Mid) for control and COVID-19 retinas were counted for vessel density manually and averaged and plotted in the graph. Total GFAP intensity measurement was performed using ImageJ. Total fluorescence for each GFAP field image (0.33 mm^2^) for control and COVID-19 retinas was calculated after subtracting the background fluorescence of the respective image and plotted in the graph for the comparison.

## Results

### Imaging of posterior globes

The combination of the fundus, confocal scanning laser ophthalmoscopy, and optical coherence tomography imaging systems can provide a comprehensive characterization of retinal lesions before histopathology [20]. Therefore, these imaging techniques were performed on all COVID-19 and control eye donors; images obtained were qualitatively compared (Fig. 1). Anatomical landmarks such as the optic nerve (ON) and fovea (Fig. 1, black arrows) were identified in all donor’s eyes using all three imaging modalities. Fundus images displayed differences between the eyes from both groups, which included several hemorrhagic spots in the COVID-19 eyes (Fig. 1A, D, and F, white arrowheads). Quantitation of these spots identified significant increased presence in COVID-19 eyes (Supplemental Fig. S1, Table). Interestingly, Case# 10, the hemorrhagic spots are similar to what has is diagnosed as roth spot hemorrhage which can be associated with systemic illnesses [21]. However, given the lack of prior clinical history for the patient, it is difficult to determine the etiology of the Roth spots in this case. SLO BAF imaging revealed several areas where the retina was detached in the COVID-19 eyes (Fig. 1H, 1J, and 1L, asterisks). However the detachment was not different between the COVID-19 and control eyes and could be a result of the processing. In addition, hemorrhagic spots in the COVID-19 eyes were observed as hypofluorescent spots (Fig. 1B, 1D, and 1F white arrowheads). Quantification of the mean pixel intensity (mean intensities in the BAF signal) of SLO images showed decreased BAF signal in the COVID-19 eyes compared to controls, but the decrease was not significant (Fig. 1M to 1P). BAF is clinically used for assessing the health of the retinal pigment epithelium (RPE). For example, there is an increase in the mean intensity or BAF signal intensity in AMD (age-related macular degeneration) patients compared with healthy controls. However, recent studies also suggest that BAF signal intensity can be used to diagnose a range of retinal disorders including but not limited to central serous chorioretinopathy, retinitis pigmentosa, hypoxia-induced damage, blockage from material anterior to RPE, intra- and retinal hemorrhages, fibrosis, and scar tissues [22, 23]. Finally, OCT-based measures for retinal thickness and optic nerve head depth showed slight increase in the COVID-19 eyes compared to controls, but the increase was not significant (Supplemental Fig. S2). In general, COVID-19 posterior poles had more vitreous debris, most likely due to detached epiretinal membranes or cellular floaters. Importantly, OCT-based measures were similar for both the OD and OS eyes of the controls and the COVID-19 donors suggesting that both eyes were equally affected (Supplemental Fig. S1S1S1).

### Histopathological and immunohistological findings in the retina of COVID-19 patients

**Fig. 2:**
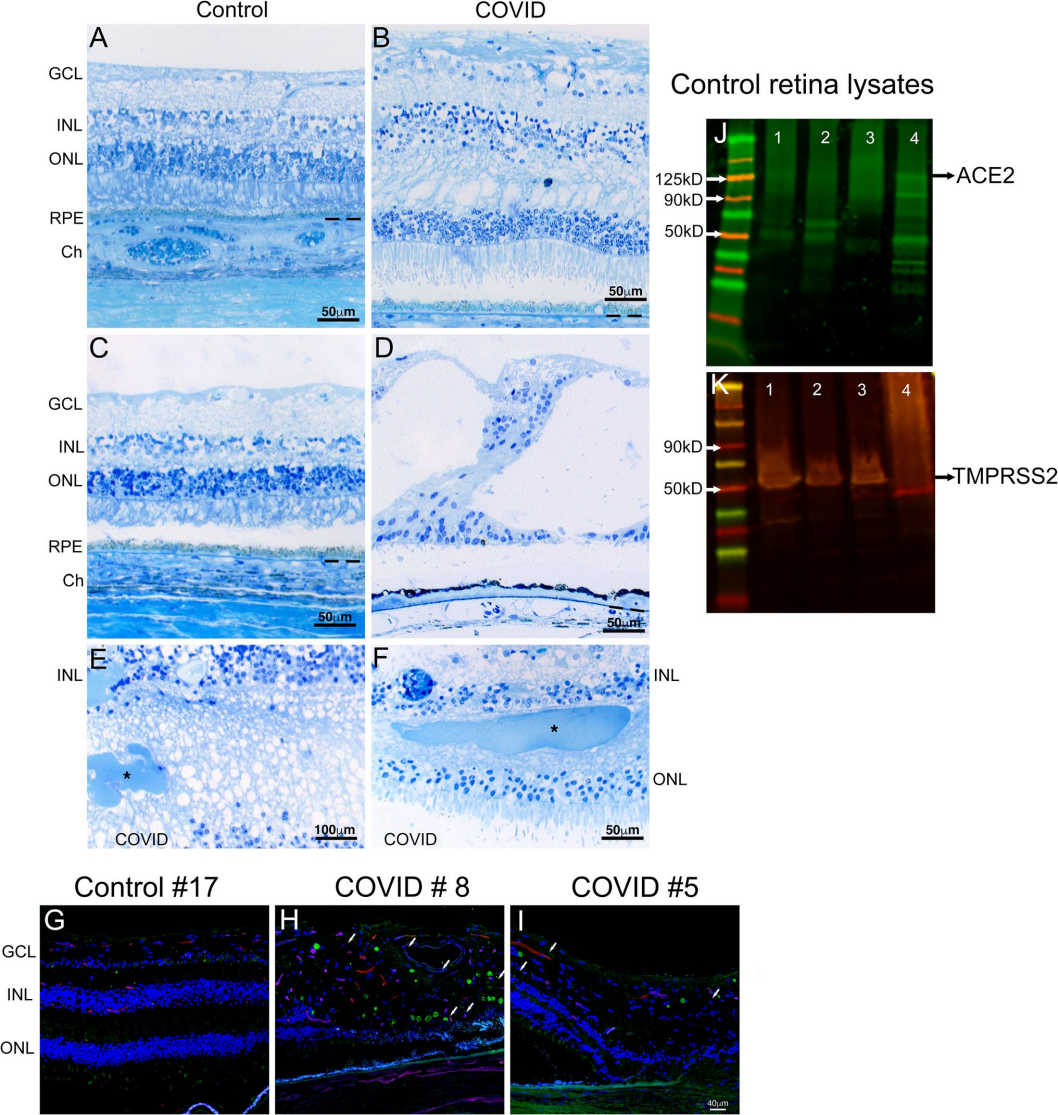
Histology and immunohistology of COVID-19 donor eyes. Representative toluidine blue-stained plastic 1 μm sections of retinas from COVID-19 donors (**B, D, E, F**) and age-similar controls (**A, C**). Morphology of the control retina displayed typical retinal lamina (**C**), while cystoid degeneration was observed in the central retina of several COVID-19 eyes (**D**). Two of the COVID-19 retinas showed hemorrhages of various sizes (**E, F**, asterisk) in the outer plexiform layer close to the optic nerve head. Immunofluorescence of two COVID-19 retinas labeled with antibodies to SARS-CoV-2 S protein (**H–I**, green) showed the presence of several positive cells (arrows) when compared to control (**G**). **J, K** Protein blots of retinal tissue from 4 healthy controls from the age group 75, probed with ACE2 and TMPRSS2 antibody indicates that these proteins are expressed in the retina. Note that the level of ACE2 is very low in control#1 and control #2, while control# 3 did not show any positive signal. TMPRSS2 is detected in the all the controls. GCL, ganglion cell layer; INL, inner nuclear layer; ONL, outer nuclear layer; POS, photoreceptor outer segments; RPE, retinal pigment epithelium; Ch, choroid. Scale bar **A**–**D**, **F** = 50 μm, **E** = 100 μm, **G**–**I** = 40 μm

Little is known about the ocular implications of COVID-19 disease. We performed a comparative histological analysis between control and COVID-19 donor eyes to determine if there are any ocular pathologies associated with COVID-19 infection. Semi-thin sections of Epon-embedded tissue were analyzed and compared to matched controls. The control donor’s retinas appear relatively normal with all the retinal layers and the RPE intact (Fig. 2A). The COVID-19 donors’ retina displayed retinal edema compared to the control retinas (Fig. 2B). Interestingly, cystoid degeneration was observed in the central area of several COVID-19 (Fig. 2D) donor's retina but not in control donors (Fig. 2C). We also observed spots of various sizes in the outer plexiform layer around the optic nerve head in a few COVID-19 donors (Fig. 2E and F, asterisks); these spots were very visible in the resin-embedded sections. However, we could not determine whether these spots were a mass of protein exudate or hemorrhagic spots. The frequency of observed morphological findings detected in our cohort is provided in Table 2. To assess whether these changes are due to the presence of the virus in the retina, an antibody that recognizes the spike protein was used to label the virus. There were several SARS-CoV-2 S positive round cells close to the optic nerve head in all the COVID-19 eyes but not in the control retinas. In two of the COVID-19-positive retinas, SARS-COV-2 S protein positive cells were detected in the retina (within the ganglion cell layer in the majority of samples and in the outer and inner plexiform layer in the samples with higher amount of labeled cells), choroid, and optic nerve head (Fig. 2H and I, arrows). The SARS-CoV-2 virus requires the angiotensin-converting enzyme 2 (ACE-2) as its receptor along with transmembrane serine protease 2 (TMPRSS2), for entry into the host cells. These proteins are present in the cornea and the conjunctival cells [7, 24]. We could also detect these proteins in the retina of healthy donors (Fig. 2J, K). Thus, it remains a possibility that some of the virus detected in the retina, could be due to a direct mode of transmission through the ocular tissue.

**Table 2:** Assessment of retinal fundus and histological findings in donor eyes

CASE N°	Hemorrhages^a^	Central Cystoid Degeneration^b^	Increased GFAP Staining	Increased infiltration of Iba-1+ cells	Central vein occlusion
2	Not Observed	X	X	X	Not observed
4	X	X	X	X	X
5	X		X	X	X^e^
6	Not observed	X	X	X^d^	X^f^
7	X		X	X	X^f^
8	X		X	X	X
10	X	X	X	X	X
11	X	Not observed	Not observed	Not observed	Not observed
12	X	Not observed	Not observed	Not observed	Not observed
13	Not observed	Not observed	Not observed	X^d^	Not observed
15	X	Not observed	X^c^	X^d^	X
16	Not observed	Not observed	Not observed	Not observed	X^f^
17	X	Not observed	X	X^d^	X^f^

^a^In at least one of the eyes

^b^Observed in both Epon-embedded sections and cryosections

^c^Slightly elevated GFAP staining observed near the optic nerve head but not towards the middle and the peripheral retina

^d^Higher numbers of Iba-1-positive cells detected in the middle and peripheral retina

^e^Vascular tortuosity in arteries and veins and aneurysms

^f^Though vein occlusion was not evident, the vascular staining was patchy in the veins and venules

### Retinal microvasculature anomalies in COVID-19 patients

**Fig. 3:**
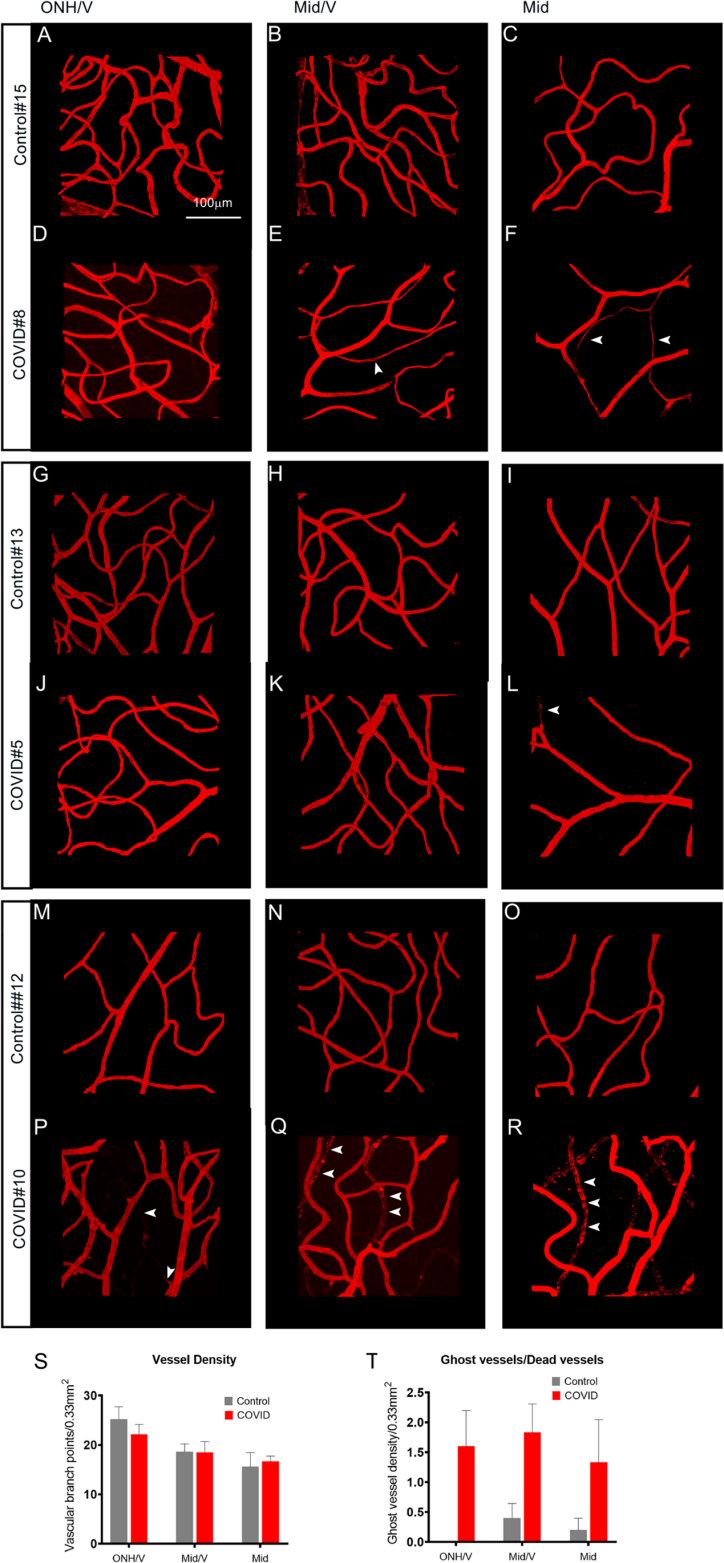
Retinal vascular abnormalities in COVID-19 patients. Representative images of retinal vasculature visualized using rhodamine-conjugated UEA lectin to label the blood vessels. **A–R** Representative images are from three different age groups. Age group 90 (**A–F**), age group 75 (**G–L**), and age group <45 (**M**–**R**). To illustrate the spatial differences in vascular density of the retinal microvasculature, the retinal preparations were subdivided into three different zones, one near the optic nerve head closer to the vein (**A, D, G, J, M, P**), one near the middle closer to the vein (**B, E, H, K, N, Q**), and one between the middle and the periphery (**C, F, I, L, O, R**). Vessel density was severely reduced together with several capillaries showing signs of atrophy(white arrowheads) in the eyes from the COVID-19 patients (**E, F, L, Q, R**) compared to age-matched controls (**B, C, I, N, O**). Most noticeable difference were observed in regions distal to the optic nerve head however in one of the COVID-19-positive sample (**P–R**); regressing vessels could be detected in the entire retina. White arrowheads indicate the severe capillary dropout. **S, T** A quantitation of the total vessel density per 0.3 mm^2^ from the different regions of all control eyes and COVID-19 eyes (**S**) and a comparison of the total number of dying capillaries in the control and the COVID-19 donor retina (**T**). ONH, optic nerve head; V, vein; Mid, middle region, Scale bar: 100 μm

There have been reports of changes in the microvasculature in patients with COVID-19. To evaluate the effects of SARS-CoV-2 infection on vasculature, we assessed both retinal and choroidal vasculature with a lectin that labels the endothelium of the vessels. There were signs of major vein occlusion in 4 out of 7 COVID-19-positive donor eyes, indicated by constriction of the vein and increased signs of hemorrhages upstream of the constrictions (Supplemental Fig. S3). Additionally, in the COVID-19-positive eyes, microvasculature density was severely reduced closer to the optic disc and around the veins (Fig. 3A to 3R) compared to the age-matched control donor eyes. Quantitation of these changes revealed a reduction in the capillary density closer to the optic nerve and the vein (Fig. 3S). In several COVID-19 eyes, there was an observable loss of microvasculature and distinct thinning of the microcapillaries compared to the control eyes (Fig. 3T). Vasculature did not appear to be different between the COVID-19 eyes and the age-matched control cohorts in the choroidal plexus. Though there was reduced focal lectin staining in some areas indicating capillary dropouts, it was difficult to determine whether there is an overall reduction in the choroidal vasculature density in the COVID-19 eyes (Supplemental Fig. S4). Nevertheless, our analysis suggests that there are distinct histological changes in the retinal microvasculature. Due to the lack of the patient’s clinical history prior to the SARS-CoV-2 infection, it is not possible to conclusively determine whether all reported vascular features can be completely attributed to the virus. However, based on the comparative analysis of the age-matched control donor eyes with similar comorbidities and with recent findings, it is highly likely that some of these retinal vascular pathologies are a consequence of the COVID-19 infection.

### Gliosis and increased infiltration of microglial cells in the retina of COVID-19 patients

**Fig. 4:**
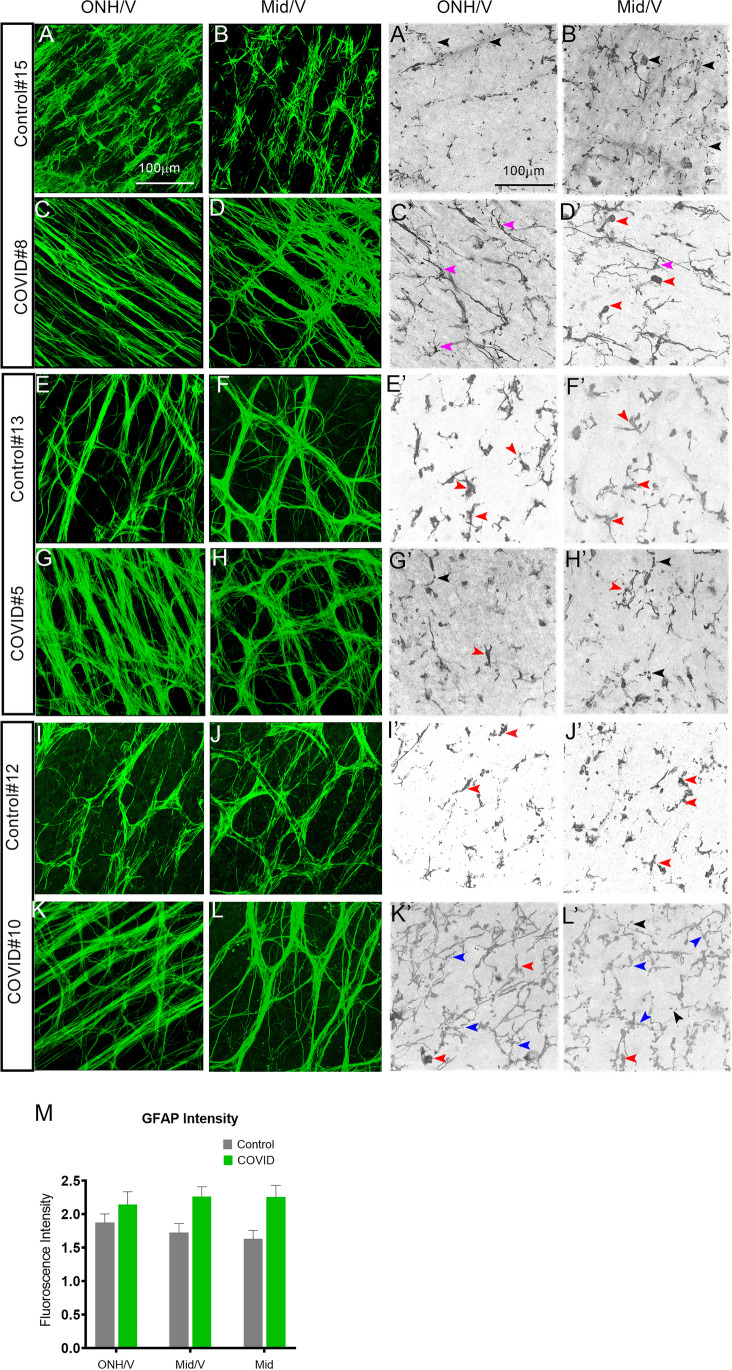
Retinal vascular anomalies are accompanied by gliosis and inflammation. Retinal whole mounts stained with an antibody for GFAP for glial cells (**A–L**; green) and Iba1 for microglia (**A’–L’**, gray). There is an overall increase in GFAP immunoreactivity near the ONH regions (**G, K**) in the COVID-19 patients when compared to age-matched controls (**E, I**). However, in one COVID-19-negative case (**A, B**), there is a similar increase in GFAP immunoreactivity near the ONH area (**A**), but not near the middle regions (**B**). In general the GFAP-positive cells appear to form dense networks and overlapping cable like structures indicative of increased gliosis. **M** Quantitation of the total GFAP intensity (as an indication of increased immunoreactivity) near the different regions of the retina from the COVID-19 and control donors. **A’–L’** Representative images of retinal tissues stained with Iba1 to visualize the microglial cells. In all the COVID-19-positive eyes, in the regions distal to the optic nerve and right next to the vein (**C’, D′, G’, H′, K′, L’**), there is an increase in the total number of microglial cells irrespective of the age of the deceased patient compared to the control eyes (**B′, F′, J’**). In general, in all of the eyes examined, several of the microglial cells displayed hypertrophic morphology (red arrowhead, **D′–L’**) with some showing fragmented processes (black arrowhead **A’, B′, G’, H′, L’**),beady, processes (blue arrowheads, **K′, L’**) indicative of dystrophic microglia with very few normal looking microglia only seen in one eye examined (magenta arrowheads, **C′, D′**). ONH, optic nerve head; V, vein; Mid, middle region, Scale bar: 100 μm

SARS-CoV-2 infection can lead to a multisystem inflammatory syndrome. Ocular tissue, like many other tissues, can be affected by this inflammatory process. Retinal and choroidal preparations were labeled with GFAP, a marker for astrocytes (Fig. 4A to 4L) and Iba-1, to label the microglial cells (Fig. 4A’ to 4L’). Irrespective of whether the eyes were from COVID-19-positive or COVID-19-negative individuals, Iba-1-positive microglial cells were present in all the eyes. As a result of aging, an increasing proportion of microglial cells display abnormal morphological features such as shortened, gnarled, beaded, or fragmented cytoplasmic processes and loss of fine ramifications and formation of spheroidal swellings; these changes are designated collectively as microglial dystrophy [20]. These changes are different from what occurs during microglial activation, defined as hypertrophic microglia [20–23]. There was evidence for both hypertrophic and dystrophic microglia in all the eyes examined, though there was evidence of microglial dystrophy in more of the COVID-19 eyes than the controls (Fig. 4E’–4L’, hypertrophic are indicated with red arrowheads, while dystrophic features are indicated with blue and black arrowheads; magenta is normal morphology).

Additionally, in all the COVID-19 eyes examined, there was an overall increase in the number of microglial cells (Fig. 4C’, 4D’, 4G’, 4H’, 4K’, 4L’), closer to the optic nerve head and towards the middle and the peripheral retina. An overall assessment of the increased microglial cells is also indicated in Table 2.

Besides the differences in the numbers, microglial cells also displayed activated microglia characteristics with enlarged spheroidal morphology and retracted processes. Similar to our retinal observations, there was evidence of increased microglial cells in the choroid (Supplemental Fig. S4). A direct or systemic inflammation can result in the activation of the astrocytes along with the microglial cells. Evaluation of the astrocyte networks labeled with GFAP showed highly variable levels of GFAP immunoreactivity, with temporal differences within macular and perimacular regions. In general, closer to the optic nerve, the GFAP immunoreactivity was increased in the COVID-19 patients compared to their age-matched controls (Fig. 4M). The astrocyte networks also appear to be different, with more elongated cell processes closer to the ONH (Fig. 4C, G, K), but towards the mid and peripheral regions, these elongated bundles appear to overlap and form dense mesh like structures (Fig. 4D, H, L). The increased GFAP immunoreactivity suggests signs of increased gliosis within the retina of patients infected with SARS-CoV-2 virus. However, due to the limited number of eyes examined, it is difficult to conclusively assess the influences of other factors such as age and sex for the analysis.

## Discussion

Though the SARS-CoV-2 is primarily a respiratory tract virus, there is sufficient evidence that the virus can be detected in several other tissues like the peripheral and central nervous systems. The first step of the SARS-CoV-2 infection involves the interaction of the virus S protein with the cell surface receptor ACE2. The SARS-CoV-2 spike glycoprotein (S) protein contains an N-terminal S1 subunit (residue 14–685) and a C-terminal S2 region (residue 686–1273) essential for the viral binding to host cells [25, 26]. The S1 subunit contains a receptor-binding domain (RBD) that binds to the peptidase domain [27] of angiotensin-converting enzyme 2 (ACE2). This interaction triggers a conformational change in the S2 subunit leading to the exposure and priming through the cleavage site at the S2 subunit by cellular proteases such as cell surface transmembrane protease serine 2 (TMPRSS2) [25, 26]. After proteolysis of the S protein, the virion begins to fuse with the host cell membrane and enter the host cell through endocytosis. ACE-2 is expressed in the lung, kidney, and gastrointestinal tract, tissues shown to harbor SARS-CoV-2 [28]. Immunohistochemistry shows ACE2 protein predominantly in the coronary and intrarenal vessels’ endothelium and renal tubular epithelium [28]. TMPRSS2 protein is highly expressed in the lungs’ bronchial epithelial cells than in surfactant producing type II alveolar cells and alveolar macrophages [29]. Previous report detected ACE2 protein in both nondiabetic and diabetic retina from human donors [27]. Another study reported immunolocalization of ACE2 predominantly to the inner nuclear layer (INL) and photoreceptors [7]. The presence of the ACE2 receptor and the cofactor TMPRSS2 in several anatomical parts of the anterior surface suggest that the SAR-CoV-2 infection of the eye tissues, especially at the limbus and the cornea, is possible. In agreement with this observation, a recent report estimated the prevalence of ocular manifestations in COVID-19 patients to range between 2 and 32% [30]. The ophthalmic manifestations appear to be associated with the disease severity of COVID-19 [1, 31]. Several groups have reported varying viral RNA and protein levels within the tears, conjunctiva, cornea, and vitreous [11, 32]. The viral ribonucleic acid [33] of SARS-CoV-2 was reported in three of fourteen human retinas of deceased patients with confirmed COVID-19 by real-time reverse transcriptase-polymerase chain reaction (RT-PCR) [34]. In this study, SARS-CoV-2 Spike protein immunoreactivity showed different degrees of distinct and specific localization in round cells within the retina of all the COVID-19 eyes close to the optic nerve head. Additionally, our results show that both ACE2 receptor and TMPRSS2 protein are present in the retinal tissue of control donors. Thus, it remains a possibility that the mode of transmission for the virus could be through the direct infection of the ocular tissue. The anterior segments of our COVID-19 cohort’s eyes were previously analyzed [11]. Of the eleven eyes recovered from seven COVID-19 donors: three conjunctival, one anterior corneal, five posterior corneal, and three vitreous swabs tested positive for SARS-CoV-2 RNA. SARS-CoV-2 can cause anterior segment pathologies such as conjunctivitis and anterior uveitis and posterior pathologies, including retinitis, optic neuritis, choroiditis with retinal detachment, and retinal vasculitis [31, 35]. These studies further illustrate the importance of long-term assessments of ocular physiology of individuals that have recovered from COVID-19.

To date, there is no detailed cellular and molecular characterization of the retina in SARS-CoV-2-infected patients. Previous studies reported retinal lesions in outpatients after confirmed SARS-CoV-2 infection with mild to moderate symptoms. Findings included non-specific and controversial hyper-reflective OCT lesions in the ganglion cell and inner plexiform layers, microhemorrhages, and nerve fiber infarcts [36]. In fundus photographs, other studies identified hemorrhages, cotton wool spots, dilated veins, and tortuous vessels [13, 37]. These are commonly seen as manifestations of diabetes mellitus and systemic hypertension but are also associated with several other etiologies, including ischemic, embolic, connective tissue, neoplastic, and infectious [38–41]. In our histological study, we detected amorphous debris in the outer plexiform layer corresponding to hemorrhages. Another exciting finding observed in COVID-19 eyes was the presence of a large number of cystoid lesions in the central retina that closely resemble the retinal alterations of patients suffering from cystoid macular edema (CME) [42, 43]. CME is observed in human retinal diseases like AMD, diabetic retinopathy, retinal vein occlusion, retinitis pigmentosa [42, 44], and optic atrophy [45], among others [43, 46]. The presence of cysts causes a thickening of the affected retina and decreases visual acuity. Moreover, the neuroretina’s compression, the nerve fibers, and capillaries by the cystic alterations further contribute to retinal degeneration and aggravation of hypoxic conditions.

Though data on histopathological analysis of retinal vasculature and choriocapillaris is scarce, there have been a few recent studies on OCTA-based findings in patients with SARS-CoV-2. In a study [12], conducted 2 weeks after recovery from systemic COVID-19, OCTA-based analysis showed a decrease in mean vessel density in the COVID-19 cohort versus the age-matched controls. In another report by Turker et al. [40], a reduction in retinal vessel density of the superficial and deep capillary plexus was noted. In their study, the measurements were done within 1 week of discharge after complete recovery. Another OCTA-based study reported several retinal findings, including hemorrhages, cotton wool spots, dilated veins, tortuous vessels, and changes in mean arterial and vein diameter in patients with COVID-19 [13]. However, the authors stated that such findings indicating microangiopathy might be secondary to COVID-19 or incidental. The authors also speculated that the virus or the systemic treatments used might have triggered microangiopathy in patients with systemic vascular disease. In the present study, from post-mortem tissue, we detect a similar reduction in retinal vascular density in COVID-19 patients along with increased vessel tortuosity, vein occlusions, and hemorrhages. Unlike the retinal vasculature, the choroidal vasculature is not severely affected due to the COVID-19 infection. It is important to note that studies have found little or no change in retinal vascular density [41]. These apparent differences in reported observations could be due to the disease severity, study populations, diagnostic criteria, and methodologies used in the different studies. It remains to be determined whether the changes in the retinal vasculature are due to a direct infection of the retina or whether these are part of common systemic vascular diseases such as diabetes mellitus, chronic kidney disease, and hypertension.

We also find evidence of increased microglial cells in the retina of the COVID-19 eyes. There is no reported evidence yet for increased infiltration of inflammatory cells in the retina. However, a recent case study reported a possible association between COVID-19 and papillophlebitis, a rare condition that occurs due to inflammation of the retinal vein [42]. Chronological aging is associated with a significant increase in the total numbers of hypertrophic and dystrophic microglia. A recent study showed that dystrophic microglia are disease-associated microglia [21]. In the present study, we see evidence for increased microglial cells in the COVID-19 eyes, and several of these appear to show the characteristic of microglial dystrophy and hypertrophy. Hypertrophic microglia are associated with increased microglial activation. However, due to the low numbers of eyes examined and the subjectivity associated with classifying a hypertrophic, dystrophic, and normal microglia solely based on morphological attributes, it is challenging to infer whether these dystrophic/hypertrophic morphological features are due to changes in the microglia. There is likely more than one explanation for the differences detected in the microglial morphology; whether this is a direct consequence of the SARS-CoV-2 infection remains to be determined. However, these preliminary findings are exciting and suggest that there could be an increase in the secretion of the pro-inflammatory molecules as a result of the microglial dystrophy and warrant further investigation. Though there is no report on activation of microglia in the retina, there is some evidence of gliosis with diffuse activation of microglia and astrocytes from neuropathological findings in patients who have died from COVID-19. Our study also finds evidence of increased astrocyte activation as indicated by the increase in GFAP immunoreactivity.

The astrocyte morphology also differs, with more elongated morphology closer to the ONH and dense mesh-like networks towards the middle and the periphery. Such phenotypic heterogeneity is associated with different responses of the astrocytes to an injury and their adaptive functions. Notably, the dense mesh-like network indicates scar formation after an injury [43, 44]. GFAP is present in COVID-19 patient’s plasma, and its levels are directly correlated with the severity of the disease [45, 46]. Whether the observed activation is temporary and is resolved after the infection is not known. However, even a short-term increase in GFAP can be detrimental to the underlying neuronal cells and result in focal damage. Despite the small sample size, the work presented here raises the possibility of subclinical vascular deficits combined with increased inflammation in patients with severe disease who have recovered from COVID-19 infection.

This study has some limitations, including the small sample size and the broad inclusion criteria. The severity of the viral infection is unknown, and the duration of hospitalization could severely impact the histopathological findings, thus limiting the generalization of the results. Due to the small numbers of available control eyes, the cause of death for these control cohorts will have a significant impact on all the reported assessments. Additionally, all histological evaluations are reported from one eye due to the limited tissue availability. Further, the treatment of the eye with the disinfectant PVP-I could impact the detection of the virus, as has been noted by Sawant et al. In this study, as stated, we used the OD eyes for the analysis. These eyes were recovered without the PVP-I exposure. It could explain why the viral spike protein immunoreactivity is detected in all the COVID-positive retina. However, the fundus and OCT analysis show no difference between the OD and OS eyes in control or the COVID-19-positive donors (Supplement Fig. S1). Though unlikely, it remains possible that the severity of phenotypes could be different between the two eyes. All care was taken to ensure that the investigated cohorts were closely matched in terms of age and the duration that these patients were maintained on ventilators. These limitations do not change the results since the prevalence of these findings was higher in the deceased patients with COVID-19 compared to the healthy controls. Further evaluation with much larger sample size is needed to determine the effects of SAR-CoV-2 infection on retinal morphology, vasculature, inflammation, and gliosis.

In conclusion, we observed several ocular anomalies in the COVID-19 cohorts compared to the control cohorts. Surprisingly, despite the small sample size, we detected consistent differences between the patient cohorts and the COVID-19-negative patients. Of note are the subclinical microvasculature features that we observed. As some of these observations have not been noted previously, our analysis suggests that as more individuals recover from the COVID-19 infections, depending on the severity of their illness, these individuals may present with clinical ocular symptoms that have not been examined previously. Therefore, a heightened vigilance for long-term disease sequelae in other tissues like the eye is warranted.

## Supplementary information

ESM 1 (PDF 1361 kb)
