# Supplementary material for: Histopathological assessments reveal retinal vascular changes, inflammation, and gliosis in patients with lethal COVID-19
Source: Graefes Arch Clin Exp Ophthalmol. 2021 Oct 29;260(4):1275–88. doi: 10.1007/s00417-021-05460-1 (PMC8553591; doi:10.1007/s00417-021-05460-1)
Supplement: Supplementary file 1 — (PDF 1361 kb) [file 417_2021_5460_MOESM1_ESM.pdf]

Figure S1

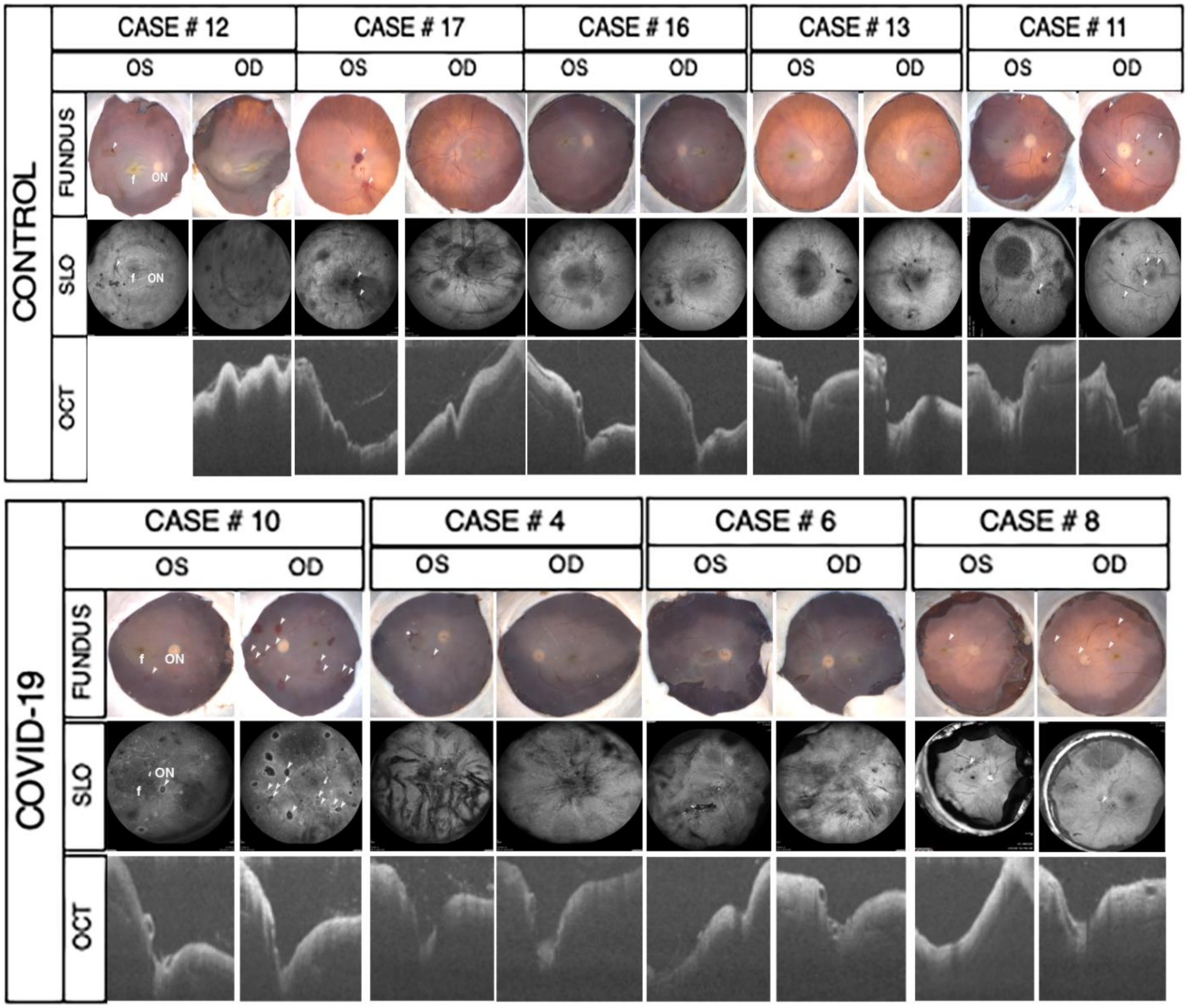

Case #	Number of Hemmorhage spots		SARS-CoV-2
	OD	OS	
2		3	Positive
4	3	6	Positive
5	9		Positive
6	4	10	Positive
7	3		Positive
8	13	11	Positive
10	13	7	Positive
11	10	8	Negative
12	2	5	Negative
13	3	1	Negative
15		3	Negative
16	0	1	Negative
17	0	7	Negative

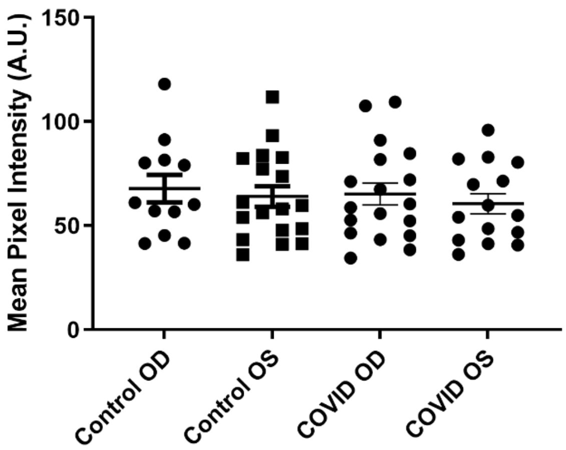

Fig. S1 Ex vivo imagings of the OD and OS globes from control and COVID 19: The top panel shows the fundus images with corresponding fundus autofluorescence images acquired using blue autofluorescence mode of scanning laser ophthalmoscopy (SLO) and the last panel indicates the Optical coherence tomography (OCT) cross sectional images with a representative B-scan of the optic nerve head. ON is optic nerve head, F is Fovea, white arrow heads indicate Hemorrhage spots. Graph indicates the quantitation of the mean signal intensity of the BAF calculated for the OD and OS. BAF intensity was similar between the two eyes. Table shows the number of Hemmorhage spots between the two eyes. On average the number of hemorraghe spots are higher in the COVID eyes compared to the controls.

Figure S2

Age group 90
Control #15 COVID # 8

Age group 75
Control #13 COVID #5

Age group <46
Control # 12 COVID #10

A

B

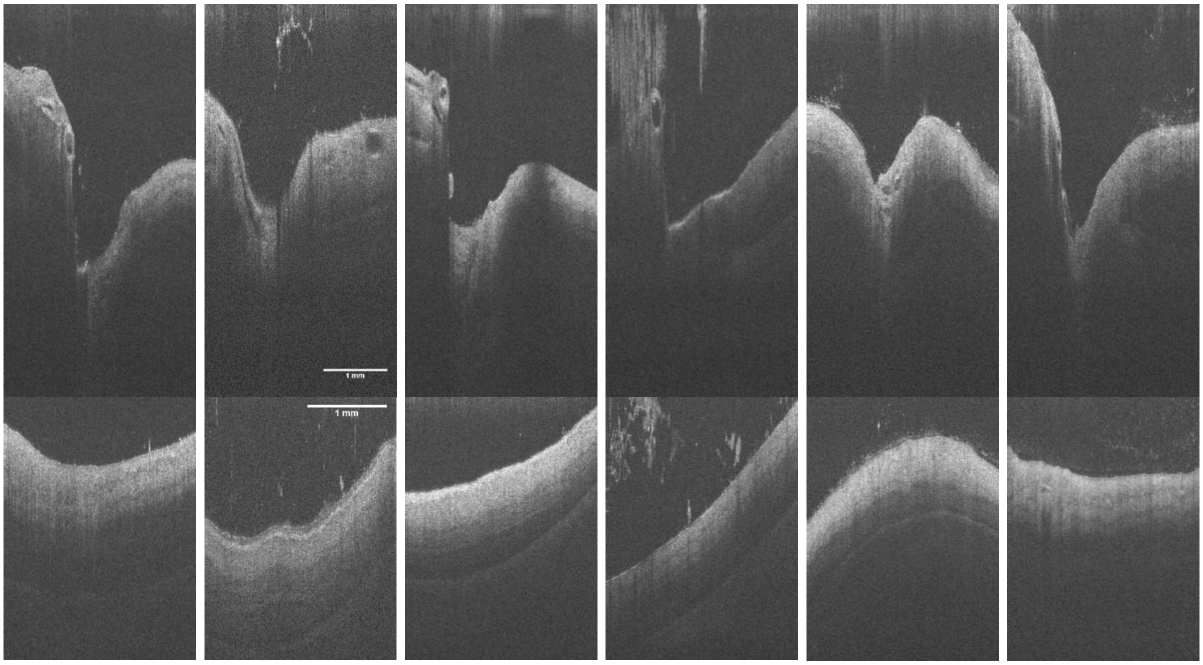

1 2 3 4 5 6

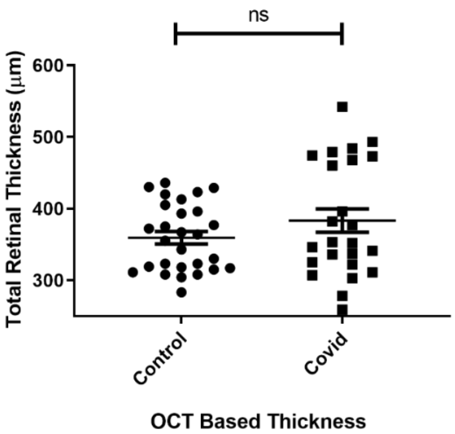

C

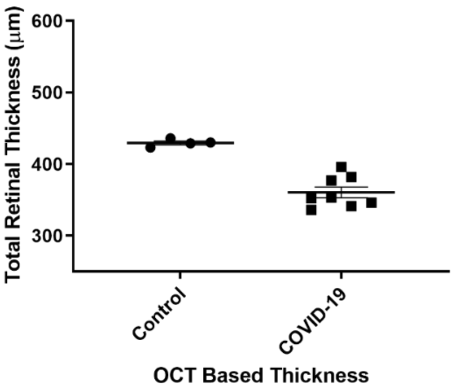

D

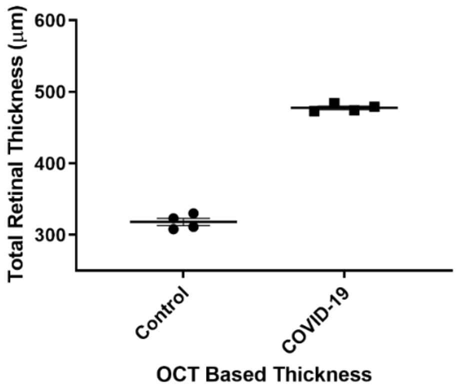

E

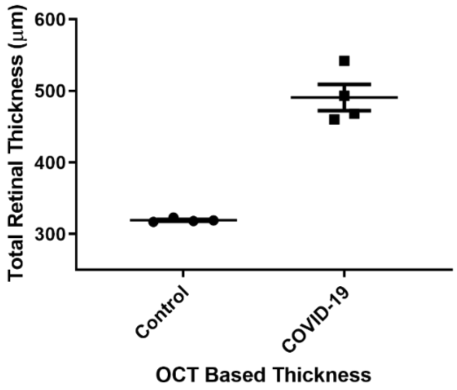

F

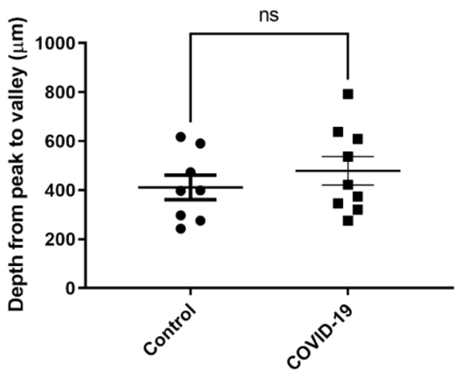

G

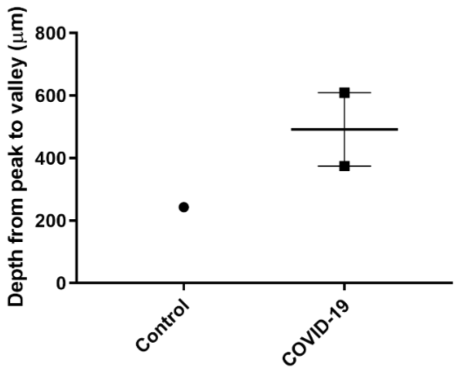

H

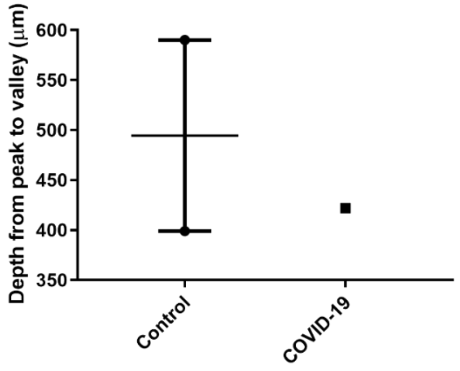

I

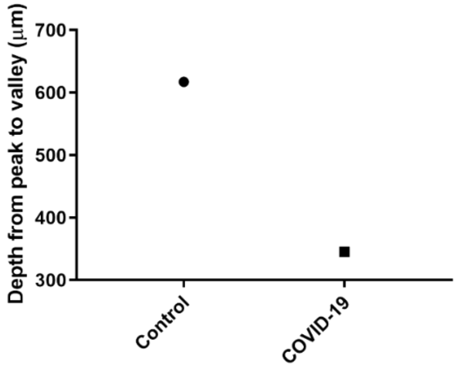

J

Fig. S2 Optical coherence tomography (OCT) cross sectional images of COVID-19 and age match control eyes, with a representative B-scan of (A) optic nerve head (ONH) and (B) central retina. The three age groups, 90, 75, and <46 were compared for retinal integrity and ONH cup size. On average, the ONH cup size was smaller in COVID-19 eyes in groups 90 and 75 when compared with control eyes. The retina layers integrity had no visible differences between the groups. Scale bar = 1 mm. Averaged total retinal thickness (RNFL to RPE) using OCT based retinal b-scans, with (C) All age groups, (D) age 90, (E) age

75, and (F) age <46. The difference was not significant between the two groups in all age category, and statistical test was not performed on remaining groups because of less number of samples. The average total retinal thickness (measured at four location, each 200 µm apart, on the b-scan) was slightly increased in COVID-19 but was not statistically significant (i.e. $P<0.05$, in a two-tailed parametric t-test). Optic nerve head depth measurement (from peak to valley) using OCT images, with (G) All age groups (H) age group <46, (I) age group 75, and (J) age group 90. On average, the difference was not statically significant between the two groups in the all age group comparison (G).

Figure S3

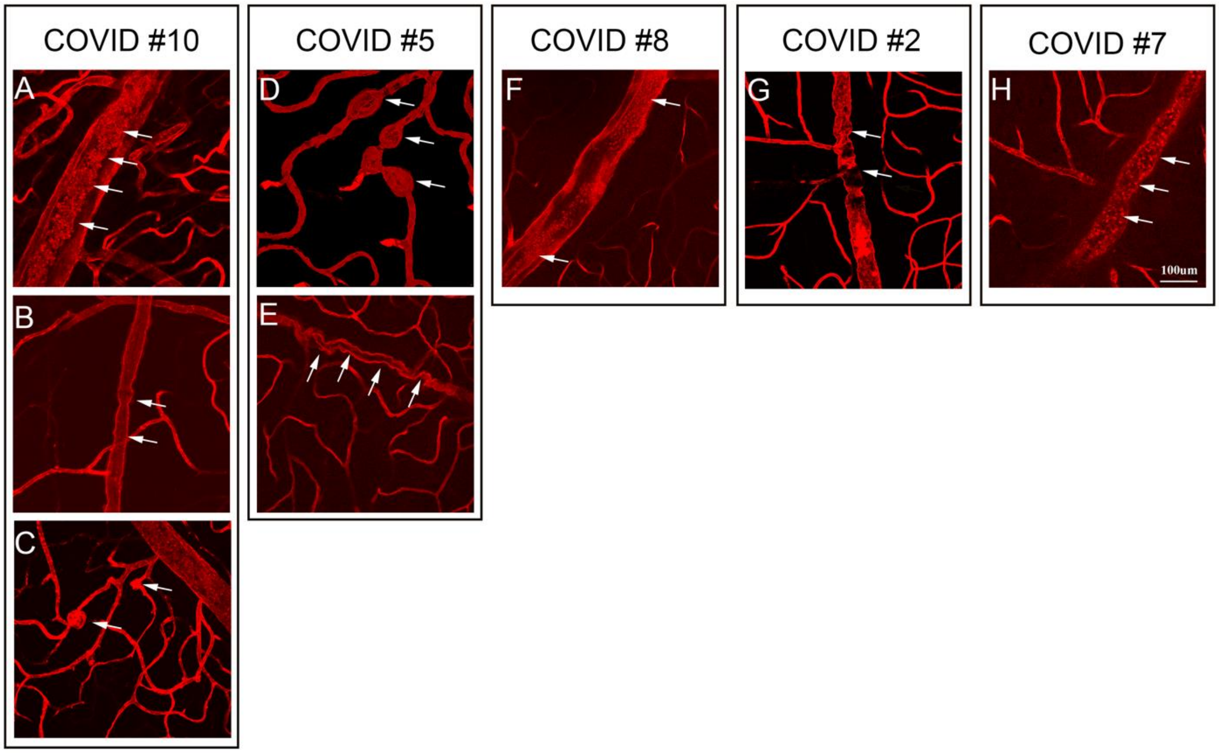

Fig. S3 Detection of other vascular abnormalities in COVID-19 patients after staining with lectin: (A, F, H) Representative images of the retinal vein occlusion, (B, E, G) vessels show marked tortuosity, (C, D) signifies vessel wall thickening which is indication of retinal arterial macroaneurysms in the COVID-19 patients. Scale bar:100 µm.

Figure S4

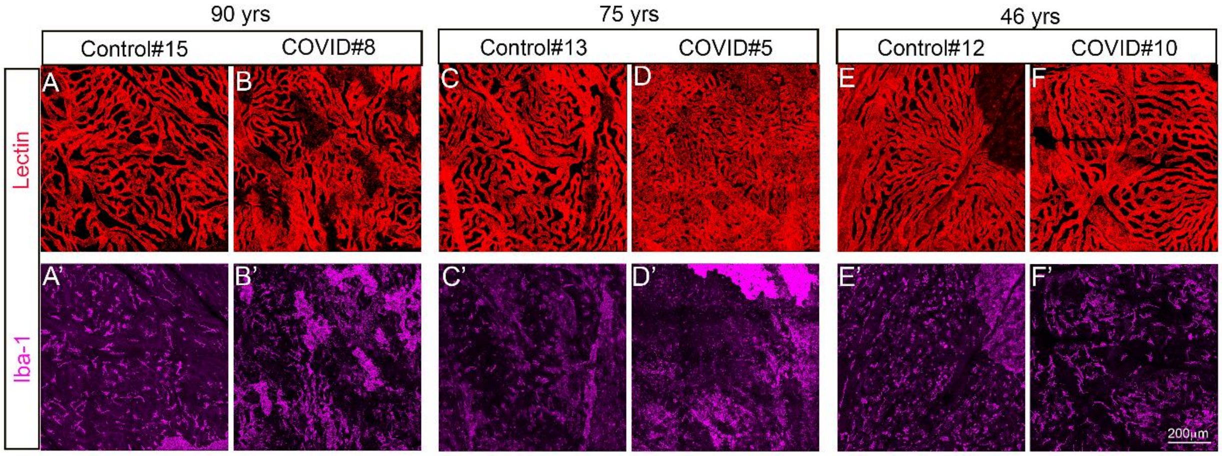

Fig. S4 Choroidal vasculature and microglial cells from COVID-19 and control cohorts (A-F'). Representative images (A-F) labeled with Rhodamine-UEA-Lectin (red) and (A'-F') Iba1 (Magenta). Choroidal vascular density appears to be similar in the COVID (B, D. F) and non-COVID individuals (A,C,E). Increase in Iba-1+ cells in the COVID-19 samples (B', D',F') was observed, although the increase is not consistent throughout the choroid. Scale bar = 200μm.
